# Pseudolaric acid B induces G2/M phase arrest in canine mammary tumor cells by targeting CDK1

**DOI:** 10.3389/fvets.2025.1644200

**Published:** 2025-10-09

**Authors:** Mengjuan Chen, Hui Han, Mengke Qin, Huixin Li, Qiqi Lu, Xin Huang, Qingda Meng, Shanshan Xie

**Affiliations:** 1 College of Veterinary Medicine, Henan Agricultural University, Zhengzhou, China; 2 Research Center for Organoids, College of Veterinary Medicine, Henan Agricultural University, Zhengzhou, China

**Keywords:** canine mammary tumor, Pseudolaric acid B, CDK1, cell cycle, cell apoptosis

## Abstract

**Introduction:**

Current management of canine mammary tumors (CMTs) remains reliant on surgical resection and chemotherapy. However, these strategies are often limited by high recurrence rates and systemic toxicity. Addressing these limitations requires urgent development of safer and more effective therapeutics. Pseudolaric acid B (PAB), a bioactive compound extracted from the roots of the *Pseudolarix kaempferi* Gord., has garnered attention for its broad-spectrum antitumor activity and favorable pharmacokinetic profile, and it has shown promise in inhibiting the growth of a variety of tumors, including breast cancer. The aim of this study was to investigate the anticancer effects of PAB on canine mammary tumor U27 cells and its underlying mechanisms.

**Methods and results:**

*In vitro* analyses demonstrated that PAB dose dependently reduced cell viability, suppressed cell proliferation, and triggered caspase-mediated apoptosis. Transcriptomic profiling of PAB-treated tumor cells revealed significant enrichment of differentially expressed genes in pathways such as gap junction, cell cycle, and cellular senescence. Mechanistically, CDK1 suppression by PAB, achieved through binding that diminishes its expression and stability, induced G2/M phase arrest and halted mitotic progression. While these findings suggest the potential of PAB as a candidate for canine mammary tumor treatment, further investigations are warranted to delineate its precise *in vivo* targeting specificity and pharmacodynamic interactions.

**Discussion:**

These findings not only expand the translational applicability of PAB in veterinary oncology but also identify CDK1 as a potential therapeutic vulnerability for combinatorial treatment strategies in CMTs.

## 1 Introduction

Canine mammary tumors (CMTs) rank as the second most prevalent malignant neoplasms following skin tumors in dogs, and represent a substantial threat to canine health (1). Surgical resection remains the primary therapeutic intervention for CMTs (2). However, even after complete excision, residual tumor microenvironment components may contribute to disease recurrence. CMTs share remarkable pathological and molecular similarities with human breast cancer, particularly in terms of molecular pathogenesis and metastatic behavior (3), making them a highly relevant translational model for anticancer drug development. Although chemotherapy and targeted therapies have improved overall survival and reduced mortality in human breast cancer patients (4), approximately 30% eventually develop therapeutic resistance, compounded by substantial drug-related adverse effects. These limitations underscore an urgent unmet clinical need for the identification of novel, more effective, and safer therapeutic targets, pharmacological agents, and bioactive compounds.

Naturally derived small-molecule compounds with well-defined structures and demonstrated therapeutic efficacies, have long been regarded as promising candidates for anti-cancer drug development (5, 6). Pseudolaric acid B (PAB), an acid isolated from the root of *Pseudolarix kaempferi* Gorden, has broad-spectrum biological properties, with potent anti-fungal, anti-virus, anti-angiogenic, and anti-fertility activities (7–9). Furthermore, PAB exerts anti-tumor efficacy against various malignancies, particularly cervical, gastric, and breast cancers (10–12). Mechanistic studies reveal that PAB induces G2/M phase cell cycle arrest and subsequent apoptosis through modulating the ROS-triggered AMPK/mTOR, PI3K/AKT, ERK1/2 and Wnt signaling pathways (7, 10, 12). However, the direct molecular target(s) of PAB in breast cancer cells and its precise mechanism of action remains to be fully elucidated.

CDK1 (Cyclin-Dependent Kinase 1), a member of Cyclin dependent kinases (CDKs) family, is a highly conserved Ser/Thr protein kinase (13). CDK1, one of the most extensively characterized cell cycle regulators, serves as the master kinase coordinating critical proliferation pathways (14, 15). Its complex with Cyclin B1 constitutes the essential driver of mitotic entry, predominantly controlling the G2/M transition and representing CDK indispensable for mammalian cell cycle progression (16). Studies have reported that abnormal expression and activity changes of CDK1 can promote tumorigenesis and immune escape of breast cancer through a variety of mechanisms (17–19). The deubiquitinating enzyme YOD1 constitutes a key tumorigenic driver in triple-negative breast cancer by stabilizing CDK1 (20). Selective targeting of CDK1, rather than CDK4/6 or CDK2, may exert lethal effects on MYC-dependent breast cancer cells (21). Furthermore, CDK1 exerts extracyclic oncogenic functions through substrate phosphorylation. Its targeting of USP29 stabilizes the TWIST1 transcription factor, consequently promoting EMT, cancer stemness, chemoresistance, and metastasis in triple-negative breast cancer (TNBC) (20). These findings highlight the critical role of CDK1 in breast cancer progression and its therapeutic potential in breast cancer subtypes.

Based on the above, this study systematically explored the anti-tumor effects of PAB in canine mammary tumor through *in vitro* experiments. Transcriptome sequencing analysis further elucidated the underlying mechanism of PAB and identified CDK1 as a potential therapeutic target. Our results validated that PAB suppressed cancer cell proliferation via cell cycle arrest at the G2/M phase in CMT-U27 cells, which is mechanistically linked to the downregulation of CDK1. Consequently, this research evaluated the anti-cancer effect and molecular mechanism of PAB in canine mammary cancer, providing an effective, low-toxicity drug and a new therapeutic strategy for the clinical treatment of breast cancer.

## 2 Materials and methods

### 2.1 Cell culture and drug treatment

Canine mammary tumor U27 cells were obtained from OriCell (Guangzhou, China), and cultured in RPMI 1640 medium (#10491, Solarbio, Beijing, China) supplemented with 10% fetal bovine serum (FBS) (#FBSST-01033-500, Oricell, Guangzhou, China), 1% penicillin–streptomycin (P/S) (#P1400, Solarbio, Beijing, China) at 37 °C in 5% CO_2_. Pseudolaric acid B, PAB (10 mM in DMSO) (#HY-N6939, MedChemExpress, USA) and Ro-3306 (10 mM) (#HY-12529, MCE, Shanghai, China) were diluted with RPMI 1640 culture medium to final concentrations of 0.625, 1.25, 2.5, 5, 10, and 20 μM. U27 cells were seeded in 96-well plates at a density 1 × 10^4^ cells/well. After reaching 70–80% confluency, cells were treated with different concentrations of the drug or an equal volume of DMSO (vehicle control) for 24 h prior to harvesting.

### 2.2 Cell viability assay

CCK-8 (Cell Counting Kit-8) assay was performed to evaluate the cell viability. After 24 h drug treatment, 10 μL of CCK-8 reagent (#C0038, Beyotime, Shanghai, China) was added to each well, followed by incubation at 37 °C for 2 h. Absorbance at 450 nm was then measured using a microplate reader (Biotek, VT, USA).

### 2.3 Crystal violet cell proliferation assay

U27 cells were inoculated into 96-well plates, drug-treated for 24 h and washed with PBS. Next, cells were fixed with 100% methanol and stained with 0.1% crystal violet (Biyuntian Biotechnology Co., Ltd., Shanghai, China) for 30 min in dark. Images were captured with microscope (Sunny Optical Technology Co., Ltd., Ningbo, China). After solubilizing in 10% acetic acid, absorbance at 570 nm (OD570) was then measured using a microplate reader (Biotek, VT, USA).

### 2.4 Annexin V-FITC/PI double staining assay

Apoptosis was analyzed using an Annexin V-FITC/PI staining kit (#KTA0002, Abbkine, Wuhan, China). Cells treated with PAB were collected and washed with cold PBS. Subsequently, the cells incubated with Annexin V-FITC and PI for 15 min at room temperature under dark conditions. Apoptotic cells were analyzed using BD FACSCelesta™ flow cytometry (Becton, Dickinson and Company, NJ, USA), and the data were processed using FlowJo™ V10 software.

### 2.5 Western blot analysis

Canine mammary tumor U27 cells were seeded into 6-well plates at a density of 1 × 10^6^ cells per well. Following seeding, cells were treated with PAB or an equivalent concentration of DMSO (vehicle control) and incubated for 24 h. The cells were lysed using RIPA buffer (Epizyme, #PC101) with protease inhibitor cocktail and the concentration was determined with the BCA kit (Epizyme, #ZJ102). Protein samples were separated on 10% acrylamide SDS-PAGE gel and the protein bands were transferred to PVDF membrane (Millipore, Bedford, MA, USA). Subsequently, 5% fat-free milk was blocked at room temperature for 1 h, and the PVDF membrane was incubated with the primary antibody overnight at 4 °C. Then washed with 1 × PBST, the membrane was incubated with secondary antibody at room temperature for 1 h. Finally, the signals were detected using an ECL reagent and captured with the amersham imagequant 800 imaging system (Cytiva, USA). *β*-actin (#GB11001-100, Servicebio, Wuhan, China) was used as an internal control. Antibodies against the following proteins were used in the experiments: Caspase 3 (#9662), Caspase 9 (#9502), Bax (#2772) and Bcl-2 (#2876), which were obtained from Cell Signaling Technology (MA, USA), CDK1 (#Ab095719) obtained from Aladdin (Shanghai, China). All the above antibody dilutions are 1:1000.

### 2.6 RNA sequencing analysis of PAB-treated canine mammary tumor U27 cells

RNA sequencing was performed by Majorbio (Shanghai, China). Canine mammary tumor U27 cells were seeded into 24-well plates at a density of 1 × 10^5^ cells per well. Following seeding, cells were treated with 2.5 μM PAB or an equivalent concentration of DMSO (vehicle control) and incubated for 24 h. Then cells were collected, and total RNA was extracted using the Trizol reagent kit (Vazyme, #PC101) according to the manufacturer ‘s protocol. The concentration and purity of the extracted RNA were detected by Nanodrop2000 (Thermo Fisher Scientific, MA, USA), the integrity of RNA was detected by agarose gel electrophoresis, and the RQN value was determined by Agilent5300 (Agilent Technologies, CA, USA). Total RNA samples were subjected to poly (A) mRNA enrichment using Oligo (dT) magnetic beads. The purified mRNA was fragmented in optimized cleavage buffer followed by double-stranded cDNA synthesis using SuperScript double-stranded cDNA synthesis kit (Invitrogen, CA, USA). Following cDNA synthesis, terminal modification steps including end-repair, phosphorylation, and ‘A’ base addition were performed in accordance with standardized Illumina’s library construction protocol. And polymerase chain reaction (PCR) amplified. The resulting cDNA libraries were sequenced using Illumina NovaSeq 6,000 by Majorbio Bio-pharm Technology Co., Ltd. (Shanghai, China). The data was processed via the online platform of Majorbio Cloud Platform.^1^

### 2.7 Real-time quantitative PCR (RT-qPCR) validation of DEGs

Canine mammary tumor U27 cells were seeded in 24-well plates at 1 × 10^5^ cells/well. After overnight attachment, cells were treated with 2.5 μM PAB or vehicle control (equivalent DMSO concentration) for 24 h. Total RNA was extracted using the Trizol reagent (Vazyme, #PC101) and reverse transcriptase reaction was performed with HiScript II Q RT SuperMix for qPCR (Vazyme, #R223-01) according to the manufacturer’s recommendations. The cDNA was diluted 1:5 in nuclease-free ddH2O. Real-time PCR was conducted with SYBR Green qPCR Master Mix (Vazyme, #Q321) on a Bio-Rad CFX96 Touch™ Real-Time PCR Detection System. *GAPDH* gene was used as an internal control. All primers employed for qPCR experiments are detailed in Table 1.

**Table 1:** List of primers.

Gene name	Sequence (5′ to 3′)	Product size (bp)
*FOS*	F: CAAGCGGAGACAGACCAACT	105
	R: GTGAGCTGCCAGGATGAACT	
*NR4A1*	F: TAAGGGCTTCTTCAAGCGCA	145
	R: TTCCTTCACCATGCCTACGG	
*GAPDH*	F: GTCCCCACCCCCAATGTATC	128
	R: GTGTAGCCCAGGATGCCTTT	
*CDK1*	F: TCCATCCCTCCTGGTCAGTT	173
	R: GCAAGGCCAAAATCAGCCAA	
*PLK1*	F: AGAACCCAATGTCTGAGCGG	115
	R: GCATTGACGCTGTGTAGCTG	
*BIRC5*	F: ACCGCGTCTCTACGTTCAAG	114
	R: CCAAGTCTGGCTCGTTCTCA	
*CDCA5*	F: AAAGAGGCTTGGGTTCCCTG	95
	R: AGTGTCCAGCATCTTCCTGC	
*RB1*	F: CTCTCACCTCCTGCATTGCT	104
	R: ATCCGTGCACTCCTGTTCTG	
*P53*	F: GAGTTCGTGACCGAGGTTGT	116
	R: TTGGCCCGCAAATTTCCTTC	

### 2.8 Molecular docking analysis

The three-dimensional structure of PAB was derived from PubChem database,^2^ while the crystal structure of CDK1 was sourced from Protein Data Bank (PDB ID: 5LQF), The docking experiment between PAB and CDK1 was then performed using the Autodock Vina program, analyzing the binding energy between the ligand and the protein. The interactions were visualized using Pymol software.

### 2.9 Cell cycle analysis

For cell cycle analysis, the PAB-treated cells were collected, and fixed by 4% paraformaldehyde at room temperature. After treated with 0.2% Triton X-100 for 5 min, cells were washed with PBS, and incubated with DAPI (#C0065, Solarbio, Beijing, China) for 5 min in the dark. Then, BD FACSCelestaTM flow cytometry (Becton, Dickinson and Company, NJ, USA) was used for detecting.

### 2.10 Cellular thermal shift assay (CETSA)

In this procedure, U27 cells were exposed to PAB (2.5 μM) for 24 h before resuspended in PBS containing protease inhibitors (#HY-K0010, MCE, Shanghai, China). Both vehicle control and PAB-treated cells were systematically allocated into six experimental groups, with each group exposed to a defined thermal gradient ranging from 42 °C to 67 °C at 5 °C intervals. Following a 3-min incubation at each temperature, cells were lysed by freeze–thaw cycles in liquid nitrogen. The supernatant collected after centrifugation was mixed with protein loading buffer (#LT101S, Epizyme, Shanghai, China) and boiled for 10 min prior to analysis by SDS-PAGE to quantify CDK1 protein expression and evaluate its thermal stability under PAB treatment.

### 2.11 Statistical analysis

All analyses were repeated in triplicate, and the data were expressed as mean ± standard deviation (SD). The measurement results were conducted statistical analysis using GraphPad Prism software. Differences between two groups were analyzed by student’s t-test, and differences between three or more groups were analyzed by one-way ANOVA. Statistical details are provided in the respective figure legends. Differences with *p* < 0.05 were regarded as statistically significant.

## 3 Results

### 3.1 PAB suppresses the viability and proliferation of canine mammary tumor cells

**Figure 1:**
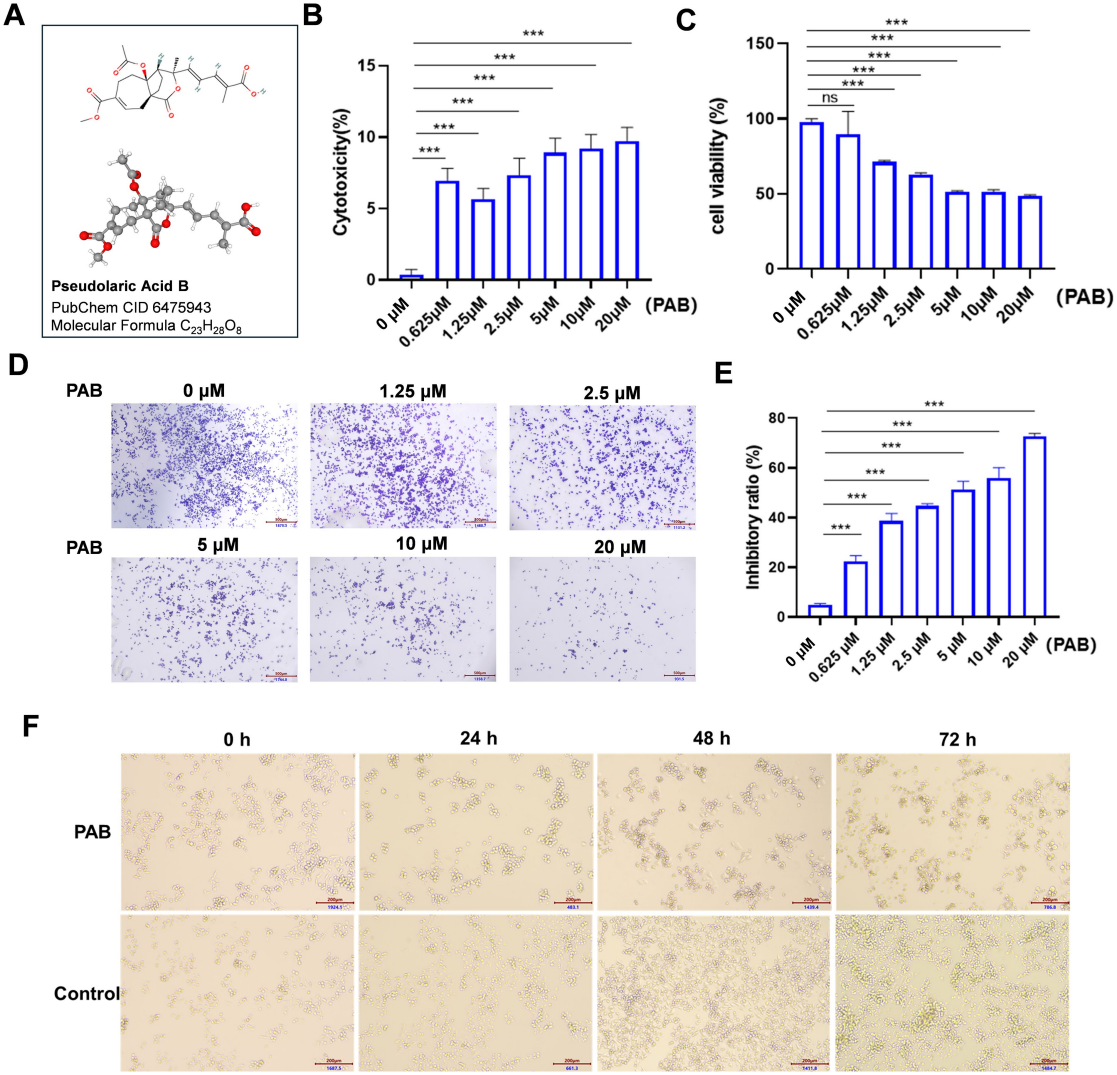
PAB inhibited the proliferation and viability in canine mammary tumor U27 cells. **(A)** Chemical structure of PAB. **(B,C)** Canine mammary tumor U27 cells were exposed to different concentrations of PAB (0, 0.625, 1.25, 2.5, 5, 10 or 20 μM) for 24 h, LDH activity **(B)** in the supernatant was quantified using a colorimetric assay by measuring absorbance at 490 nm and the cell viability **(C)** of canine mammary tumor U27 cells was detected by CCK-8 assay. **(D)** Representative images of the proliferative ability of canine mammary tumor U27 cells treated with PAB (0, 0.625, 1.25, 2.5, 5, 10 or 20 μM) via crystal violet cell proliferation assay. **(E)** Quantitative analysis of cells stained with crystal violet. Values represent mean ± SD of three biological replicates. **(F)** Representative images of morphological changes of U27 cells after PAB exposure at varying time points. DMSO-treated control cells maintained normal morphology and PAB-treated (2.5 μM) cells exhibited significant rounding. Scale bar: 200 μm. Unpaired two-tailed Student’s *t*-test or one-way ANOVA was performed. ***p < 0.001, **p < 0.01, *p < 0.05, ns, not significant.

To validate the functional effects of PAB (Figure 1A) in U27 cells, we treated them with varying concentrations of PAB (0, 0.625, 1.25, 2.5, 5, 10, and 20 μM) and investigated multiple biological processes, including proliferation and viability. We began with an LDH analysis to evaluate the toxic effects of PAB on U27 cells (Figure 1B). The results showed a highly significant cytotoxic effect (*p* < 0.001) in both low- and high-dose PAB-treated groups compared to the control group, indicating a significant loss of membrane integrity. We then assessed the impact of PAB on U27 cell viability using the CCK-8 assay. As expected, PAB suppressed U27 cell viability (*p* < 0.001), with higher concentrations of PAB significantly reducing cell viability (Figure 1C). Furthermore, crystal violet cell proliferation assay demonstrated that PAB could inhibit U27 cell proliferation (*p* < 0.001), with higher concentrations of PAB showing greater inhibition than lower concentrations (Figure 1D). The statistical results, presented in Figure 1E, show that PAB inhibited the proliferation of canine mammary tumor cells in a dose-dependent manner. Additionally, we observed that PAB exposure induced morphological alterations in U27 cells, characterized by a distinct rounding phenotype (Figure 1F). These results suggested that PAB effectively inhibits the viability and proliferation of canine mammary tumor cells.

### 3.2 PAB triggers apoptosis in canine mammary tumor cells via caspase activation

**Figure 2:**
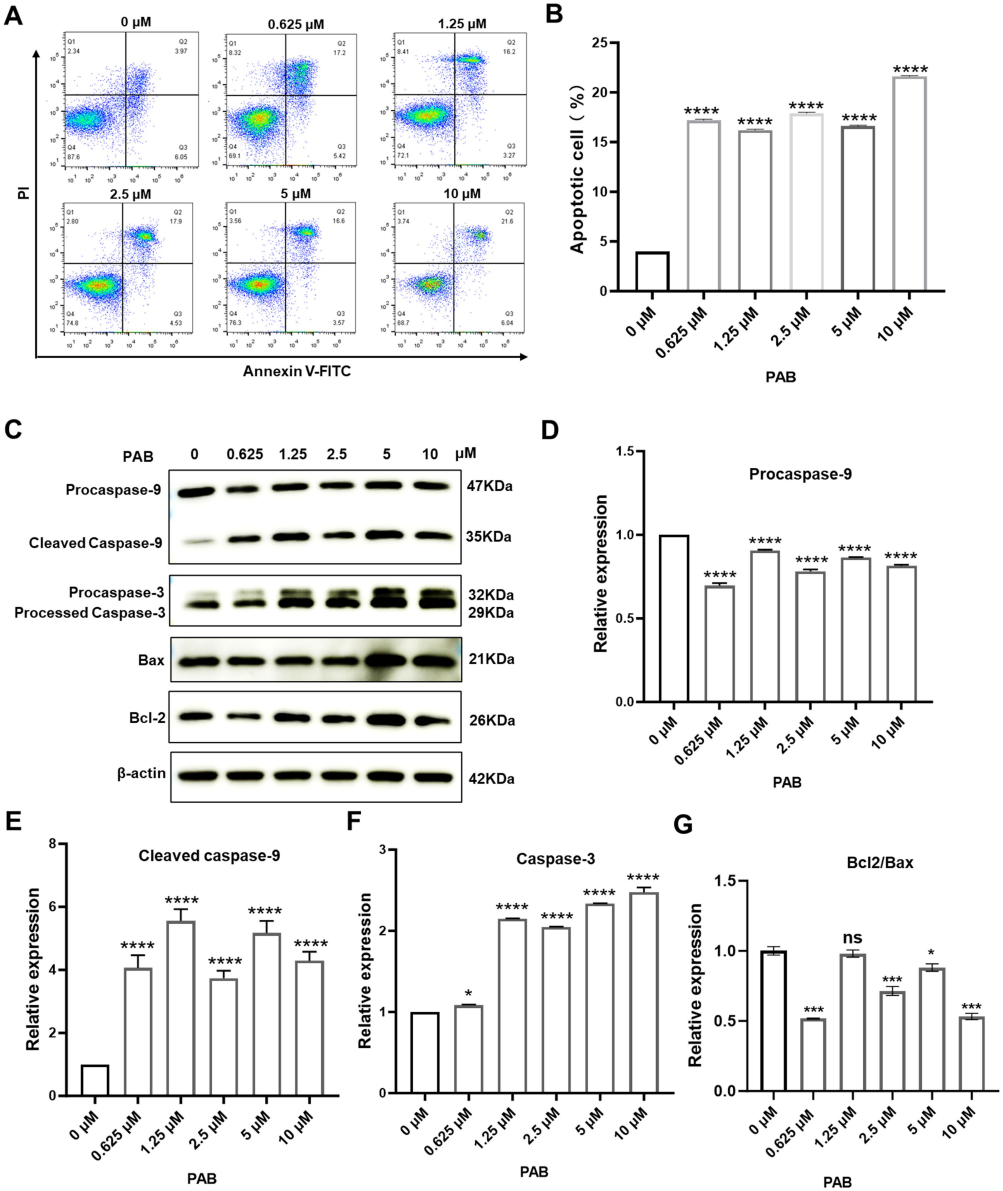
PAB triggered apoptosis through caspase activation in canine mammary tumor cells. **(A)** Flow cytometry was performed to determine the percentage of apoptotic canine mammary tumor U27 cells after 24 h of treatment with PAB (0, 0.625, 1.25, 2.5, 5 or 10 μM). **(B)** Quantitative analysis of late (end-stage) apoptotic cells (Q2). Data are presented as the percentage of apoptotic cells relative to the total population. **(C–G)** Western blot analysis **(C)** was conducted to evaluate apoptotic markers (procaspase-9, cleaved caspase-9, caspase-3, Bax and Bcl2) in canine mammary tumor U27 cells treated with PAB (0, 0.625, 1.25, 2.5, 5 or 10 μM) for 24 h. **(D–G)** Quantitative results of protein expression. *β*-Actin served as an internal reference and quantitative analysis of band intensities was performed using ImageJ software. Values represent mean ± SD of three biological replicates. Unpaired two-tailed Student’s *t*-test or one-way ANOVA was performed. ****p < 0.0001, ***p < 0.001, **p < 0.01, *p < 0.05, ns, not significant.

The induction of cancer cell death represents a fundamental objective of antineoplastic therapy, with apoptosis serving as a key mechanism for tumor suppression (22). We examined the apoptosis-inducing effects of PAB on U27 cells through flow cytometry and Western blot analysis. Flow cytometry revealed a significant increase in apoptotic tumor cells following PAB treatment (*p* < 0.001) (Figures 2A,B). To further confirm the pro-apoptotic effect of PAB on U27 cells, we analyzed changes in apoptotic marker protein expression (Figures 2C–G). Results showed downregulation of the inactive Caspase-9 precursor (pro-caspase-9) and significant increase in its cleaved, active form (*p* < 0.001). Processed caspase-3 fragment expressions were also elevated significantly (*p* < 0.001). Notably, dose-dependent upregulation of Bax (*p* < 0.001), a pro-apoptotic Bcl-2 family protein, occurred only at higher PAB concentrations (5–10 μM). Additionally, the ratio of Bcl2/Bax was downregulated after PAB treatment, indicating that the apoptotic pathway was activated. These results provide consistent evidence for the pro-apoptotic properties of PAB in U27 cells through caspase cascade activation and Bax modulation.

### 3.3 PAB alters the transcriptional landscape of canine mammary tumor cells

**Figure 3:**
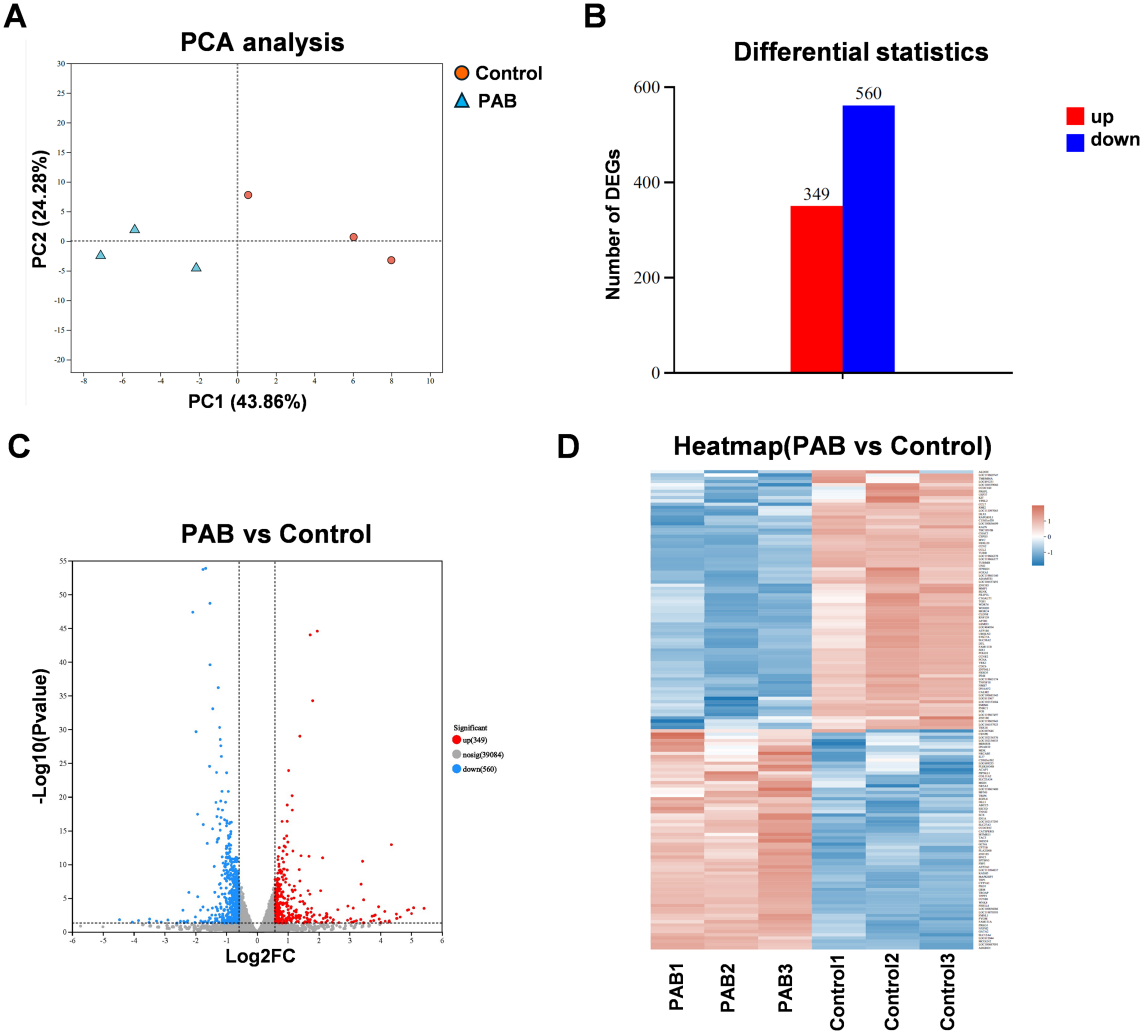
Different expression analysis in genes of canine mammary tumor cells treated with PAB. Canine mammary tumor U27 cells were seeded in 6-well plates and treated with PAB (2.5 μM) or an equivalent concentration of DMSO (vehicle control) for 24 h (*n* = 3). **(A)** PCA score plot of the transcriptomics. **(B)** Number of differentially expressed genes (DEGs) upregulated and downregulated. **(C)** Volcano plot showing DEGs of RNA-seq data. **(D)** The heatmap displayed the upregulated DEGs and downregulated DEGs ranked by fold change.

To systematically investigate PAB’s molecular mechanism of action, we conducted whole-transcriptome analysis of U27 cells following PAB or vehicle (DMSO) treatment. Principal component analysis (PCA) demonstrated clear separation between treatment groups and tight clustering of biological replicates, indicating robust experimental reproducibility and significant transcriptomic changes induced by PAB (Figure 3A). Comparative transcriptomic profiling identified 899 differentially expressed genes (DEGs) (*p* < 0.05, |log2 FC| > 1.5), comprising 349 up-regulated and 560 down-regulated transcripts (Figures 3B–D).

### 3.4 Functional enrichment of DEGs in PAB-treated canine mammary tumor cells

**Figure 4:**
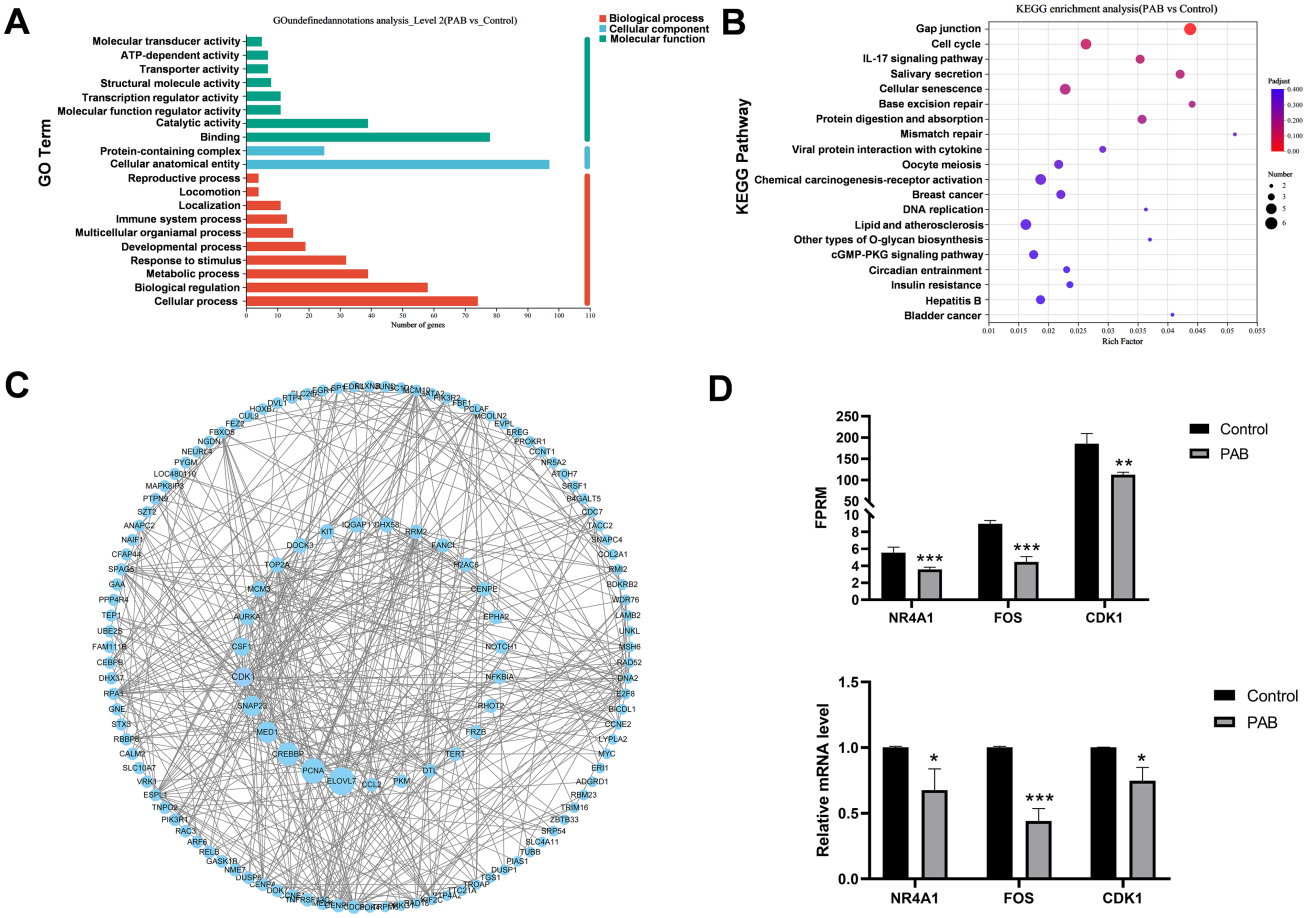
Functional enrichment analysis of DEGs in canine mammary tumor cells treated with PAB. Canine mammary tumor U27 cells were seeded in 6-well plates and treated with PAB (2.5 μM) or an equivalent concentration of DMSO (vehicle control) for 24 h (*n* = 3). **(A)** GO enrichment analysis of DEGs. **(B)** KEGG enrichment analysis of DEGs. The size of the dots represents the number of differential genes, and the intensity of the color represents the level of significance (the redder the color, the smaller the *p*-value) **(C)** PPI analysis of DEGs was performed using Cytoscape software. DEGs are arranged according to the betweenness centrality. The larger betweenness centrality, the larger the circle. **(D)** Validation of DEGs expression using RT-qPCR. *GAPDH* gene as a standardized internal reference. *N* = 3 for each group. Values represent mean ± SD of three biological replicates. Unpaired two-tailed Student’s *t*-test was performed. ***p < 0.001, **p < 0.01, *p < 0.05, ns, not significant. FPRM, Fragments Per Kilobase of exon model per Million mapped fragments.

Enrichment analysis was conducted separately for DEGs to further explore the functions of DEGs. The top 20 Gene Ontologies (GO) and the Kyoto Encyclopedia of Genes and Genomes (KEGG) pathways that were significantly enriched in each group were selected for presentation. GO term enrichment results showed that the DEGs were mainly enriched in catalytic activity, binding, cellular anatomical entity, cellular process, and biological regulation (Figure 4A). In addition, according to KEGG analysis, DEGs indicated significant enrichment in gap junction, cell cycle, cellular senescence, IL-17 signaling pathway, protein digestion and absorption (Figure 4B).

To identify potential protein targets of PAB, we performed protein–protein interaction (PPI) network analysis of DEGs, showing that *ELOVL7*, *PCNA*, *CREBBP*, *MED1*, *SNAP23*, and *CDK1* were hub genes (Figure 4C). By integrating the GTEx and TCGA databases, we analyzed the expression differences of these hub genes between human breast cancer patients and normal populations. Our analysis revealed that CDK1 gene was significantly upregulated, suggesting its potential role in breast cancer pathogenesis (Supplementary Figure S1). Notably, both RNA-seq and RT-qPCR analyses consistently demonstrated significant downregulation of CDK1 mRNA expression following PAB treatment (Figure 4D), which strongly suggests that CDK1 plays a crucial role in mediating PAB’s anti-tumor effects. To validate the RNA-seq results, we randomly selected three DEGs for RT-qPCR verification. The expression patterns of these genes were fully consistent with the RNA-seq data (Figure 4D), confirming the reliability and reproducibility of our transcriptome analysis.

### 3.5 PAB induces G2/M cell cycle arrest through CDK1 targeting

**Figure 5:**
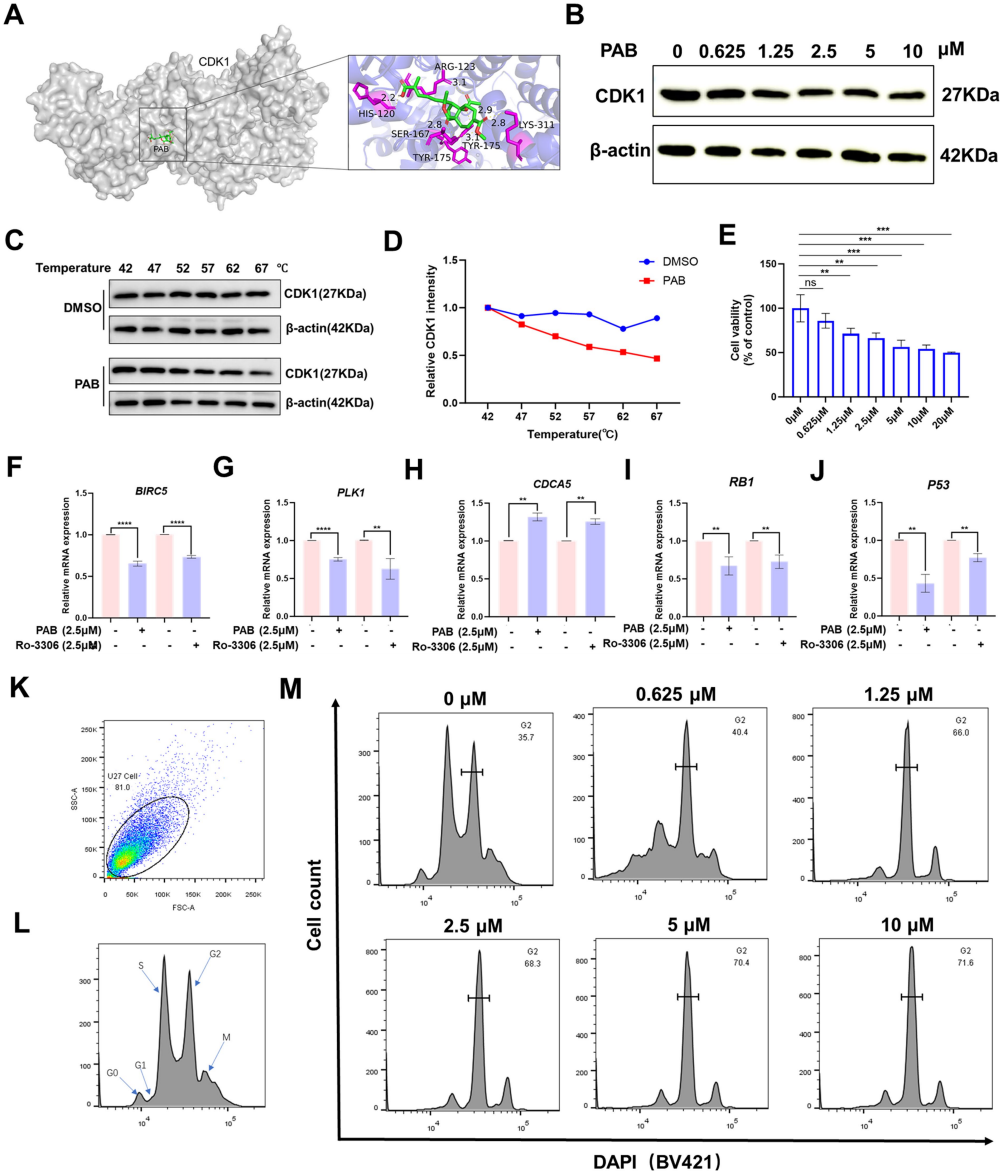
PAB induced G2/M cell cycle arrest by modulation of CDK1. **(A)** Molecular docking computer simulation predicts the binding site of PAB and CDK1 protein. **(B)** Western blot analysis of CDK1 protein expression under different concentrations (0, 0.625, 1.25, 2.5, 5 or 10 μM) for 24 h of PAB-treated canine mammary tumor U27 cells. β-Actin served as an internal reference and quantitative analysis of band intensities was performed using ImageJ software. **(C,D)** Representative western blot images of PAB (2.5 μM) and CDK1 from the CETSA (U27 cells, at 42 to 67 °C) and quantitatively analyzed using Image J software (*n* = 3). **(E)** Canine mammary tumor U27 cells were exposed to different concentrations of Ro-0036 (0, 0.625, 1.25, 2.5, 5, 10 or 20 μM) for 24 h, the cell viability of canine mammary tumor U27 cells was detected by CCK-8 assay. **(F–J)** CDK1 downstream target genes (*BIRC5*, *PLK1*, *CDCA5*, *RB1*, *P53*) mRNA expression levels were assayed after PAB or Ro-3306 treatment of U27 canine mammary tumor cells. Unpaired two-tailed Student’s t-test or one-way ANOVA was performed. **(K–M)** The cell cycle distribution of PAB-treated (0, 0.625, 1.25, 2.5, 5 or 10 μM) canine mammary tumor U27 cells for 12 h was determined by flow cytometry using DAPI staining.

Considering the important role of CDK1 in the development and progression of multiple cancers and that PAB can significantly regulate CDK1 (23), this phenomenon was subsequently investigated in depth. Molecular docking results showed that PAB was able to bind to HIS120 (2.2 Å), ARG123 (3.1 Å), LYS311 (2.8 Å), TYR175 (3.1 Å), SER167 (2.8 Å), and TYR175 (2.9 Å) of CDK1 (Figure 5A). And the calculated binding energy between PAB and CDK1 is −7.7 kJ/mol. This energetically favorable interaction was associated with significant biological effects, as evidenced by Western blot analysis showing a marked reduction in CDK1 protein levels following PAB treatment (Figure 5B). CETSA demonstrated compound-protein interactions via temperature-induced denaturation, confirming PAB binding to CDK1, which promoted thermal destabilization and degradation of CDK1 at elevated temperatures (52–67 °C) (Figures 5C,D). The concurrent demonstration of both computational binding and functional downregulation strongly supports CDK1 as a potential binding site of PAB in canine mammary tumor cells.

To further explore the properties of PAB-CDK1 interaction, we compared the effects of the CDK1 inhibitor Ro-3306 with those of PAB on canine mammary tumor cells. Similar to PAB, Ro-3306 significantly inhibited cell viability at elevated concentrations (Figure 5E). Furthermore, quantification of CDK1-dependent downstream gene expression revealed that PAB produced comparable suppressive effects to Ro-3306 (Figures 5F–J). Given CDK1’s pivotal role in controlling G2/M phase transition, whether PAB-mediated CDK1 downregulation affects cell cycle progression in U27 cells was investigated. Flow cytometric analysis revealed a dose-dependent accumulation of cells in G2/M phase following PAB treatment (66.0% at 1.25 μM vs. 35.7% in control, Figures 5K–M). This cell cycle blockade correlated with the observed reduction in CDK1 protein levels, strongly suggesting that PAB’s anti-tumor activity is mediated, at least in part, through CDK1-related inhibition of G2/M progression.

## 4 Discussion

Canine mammary tumors, predominantly affecting female dogs, represent the most prevalent neoplastic condition in this population. While the majority of canine mammary tumors exhibit benign biological behavior, approximately 50% of malignant canine neoplasms are attributed to mammary malignancies (24). Surgical resection remains the gold standard for canine mammary carcinoma treatment. While conventional chemotherapeutics (e.g., cyclophosphamide, 5-fluorouracil, doxorubicin) adapted from human breast cancer protocols have been employed in veterinary practice (25, 26). Their efficacy is often limited by dose-limiting toxicities, drug resistance, and high recurrence rates (27). Given the unlimited proliferative potential of cancer cells, the main strategy of cancer treatment is to induce cancer cell death and inhibit tumor proliferation (28). Studies have shown that PAB exerts antitumor effects through multiple pathways, including significant inhibition tumor cell growth, induction tumor cell apoptosis, and inhibition tumor invasion and migration (29). However, the molecular mechanisms of PAB in cancer remain largely unexplored. Here, the anti-tumor bioactivity of PAB was investigated *in vitro*, and PAB altered the morphology of canine mammary tumor U27 cells, inhibited cell proliferation and promoted apoptosis. The target and mechanisms of PAB action were preliminarily predicted by RNA-seq and computational simulation analysis. These findings highlight PAB’s potential as a selective therapeutic agent for canine mammary cancer, while simultaneously providing novel conceptual frameworks for advancing anticancer drug development from natural product-derived anticancer agents in veterinary oncology.

The anti-cancer activity of PAB has been studied in several cancer types. In esophageal squamous cell carcinoma, PAB could inhibit cancer cell proliferation, invasion and angiogenesis by regulating CD147 (29). In melanoma cells, PAB induces apoptosis through p53 and Bax/Bcl-2 pathways. There are also reports showing PAB may induce apoptosis dependent on the AKT/mTOR, NF-κB, p38, caspase pathways (10, 30, 31). The present study found that PAB promoted apoptosis in canine mammary tumor cells and increased the expression of Bax, caspase-3, cleaved-caspase-9, and processed-caspase-3, and that the high catalytic activity of cysteine asparaginase-3 is a common initiator of apoptotic cell death. It has been previously shown that in response to stimulation by pro-apoptotic factors, Bax proteins migrate from the cytoplasm to the outer mitochondrial membrane, altering the permeability of the outer mitochondrial membrane and facilitating the mitochondrial release of Cyt-c (32). The release of Cyt-c results in the formation of an apoptotic vesicle complex by binding Cyt-c to Apaf-1, procaspase-9 and dATP. The dimeric complex activates first caspase-9 and then caspase-3, and these activations lead to apoptosis (33, 34). These findings support the hypothesis that PAB-induced apoptosis in canine mammary cancer involves the mitochondrial pathway, though further mechanistic investigation is warranted.

To further explore the underlying mechanisms of PAB in canine mammary cancer, we performed transcriptomic sequencing. The results suggested that PAB-treated canine mammary tumor cells were involved in multiple cellular functions, including gap junction, cell cycle, cellular senescence, mismatch repair. Gap junctions are a unique form of cellular connectivity that contribute to cancer cell invasion, increase nutrient supply and waste removal within the tumor, and interact with immune cells to evade detection (35, 36). Studies have shown that PAB treatment induces L929 cell cycle blocking and induces cell aging in mouse fibrosarcoma (37). Similarly, our study found that the differential expression genes of canine mammary tumor cells after PAB treatment were significantly enriched in the cell cycle and cell aging signaling pathways. Breast carcinogenesis commonly arises from functional alterations or genetic mutations in key regulatory proteins, resulting in abnormal activation or suppression of critical signaling cascades (38). Subsequent PPI analysis of differential genes identified CDK1 as a key PAB target, with this protein playing a pivotal role in cell cycle regulation. Its expression was significantly upregulated in tumor tissue, while the expression was significantly downregulated after PAB treatment.

Previous studies have reported PAB as a cell cycle inhibitor, while CDK1 is a key mediator regulating the G2/M transition (8). PAB treatment induced cell cycle arrest at the G2/M phase in this study. Molecular docking analysis and CETSA results revealed the binding of PAB to CDK1 promoted the degradation of CDK1, reducing the thermal stability of CDK1, further supporting CDK1 as a potential functional target of PAB. Consistent with the phenomenon that senescence and cell death spontaneously occur during or following mitotic arrest in both normal and cancer cells, RNA sequencing identified that differentially expressed genes were predominantly enriched in cell cycle progression and cellular senescence signaling pathways (39). Our findings collectively reveal that PAB triggers cell cycle arrest by targeting CDK1, thereby inducing cellular senescence and apoptosis. This mechanism underscores the therapeutic potential of PAB in modulating cell fate through CDK1-dependent pathways.

**Figure 6:**
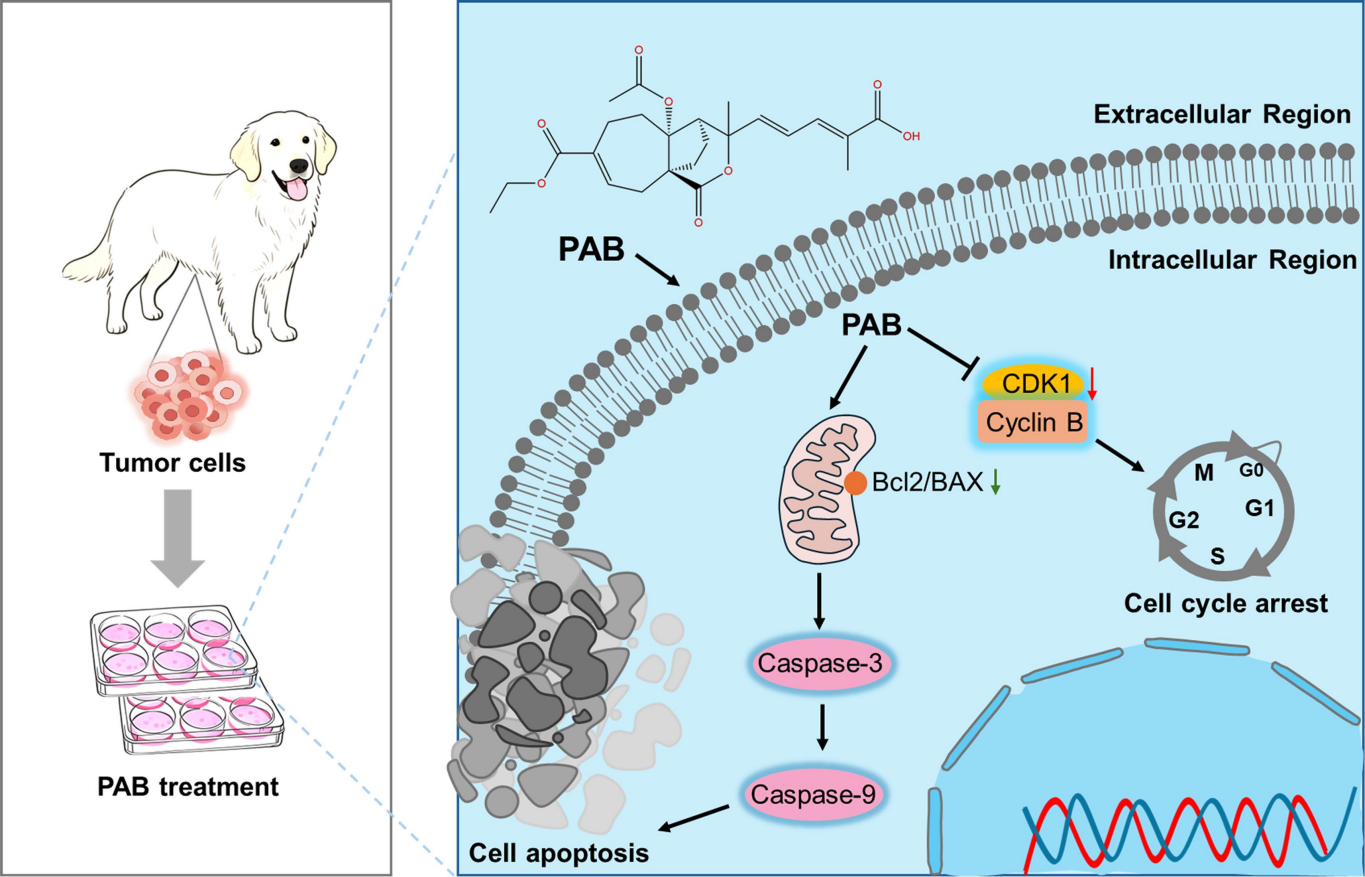
Schematic illustration of the molecular mechanism of PAB against canine mammary tumor *via* promoting tumor apoptosis and cell cycle blockade.

In summary, as a natural product-derived agent, PAB demonstrates distinct advantages over conventional chemotherapeutics by addressing critical limitations such as drug resistance and systemic toxicity through its multi-target mechanisms. Our data suggested CDK1-dependent cell cycle arrest as the central mechanism driving PAB-induced senescence and mitochondrial apoptosis (Figure 6). The compound’s ability to simultaneously target proliferative signaling and activate intrinsic apoptotic pathways addresses critical challenges in veterinary oncology, particularly tumor recurrence and chemoresistance. While these findings highlight the translational potential of PAB, certain limitations must be addressed, including *in vivo* validation of CDK1 targeting efficacy and pharmacokinetic optimization in future studies. Furthermore, comparative studies across species may elucidate conserved mechanisms to bridge veterinary and human anticancer drug development. This work not only advances natural product-based therapeutics for CMTs but also establishes a paradigm for mechanism-driven drug discovery in companion animal cancers.

## Data Availability

Sequence data that support the findings of this study have been deposited in the National Center for Biotechnology Information with the primary accession code PRJNA1290691.

## Footnotes

^1^ www.majorbio.com

^2^ https://pubchem.ncbi.nlm.nih.gov/
