# Correction: Pseudolaric acid B induces G2/M phase arrest in canine mammary tumor cells by targeting CDK1

**DOI:** 10.3389/fvets.2026.1864346

**Published:** 2026-05-14

**Authors:** Mengjuan Chen, Hui Han, Mengke Qin, Huixin Li, Qiqi Lu, Xin Huang, Qingda Meng, Shanshan Xie

**Affiliations:** 1 College of Veterinary Medicine, Henan Agricultural University, Zhengzhou, China; 2 Research Center for Organoids, College of Veterinary Medicine, Henan Agricultural University, Zhengzhou, China

**Keywords:** canine mammary tumor, Pseudolaric acid B, CDK1, cell cycle, cell apoptosis

In the published article, there was a mistake in the **Funding** statement.

The funder Natural Science Foundation of Henan, Grant No. 252300421421 to QM and Grant No. 252300420679 to SX, was erroneously omitted.

The revised **Funding** statement reads:

“The author(s) declared that financial support was received for this work and/or its publication. This work was supported by the National Natural Science Foundation of China (Grant No. 32502996) to SX; the Ministry of Human Resources and Social Security of China (Grant No. 109-23XM0447) to QM; the Natural Science Foundation of Henan (Grant No. 252300421421 to QM and Grant No. 252300420679 to SX); and the High-level Talent Support Program of Henan Agricultural University (Grant Nos. 111-30501275 and 111-30501434) to QM and SX.”

The original version of this article has been updated.

