# Supplementary material for: Pseudolaric acid B induces G2/M phase arrest in canine mammary tumor cells by targeting CDK1
Source: Front Vet Sci. 2025 Oct 9;12:1644200. doi: 10.3389/fvets.2025.1644200 (PMC12547503; doi:10.3389/fvets.2025.1644200)
Supplement: Supplementary file 1 [file Data_Sheet_1.DOCX]

**Supplemental Figure 1.**


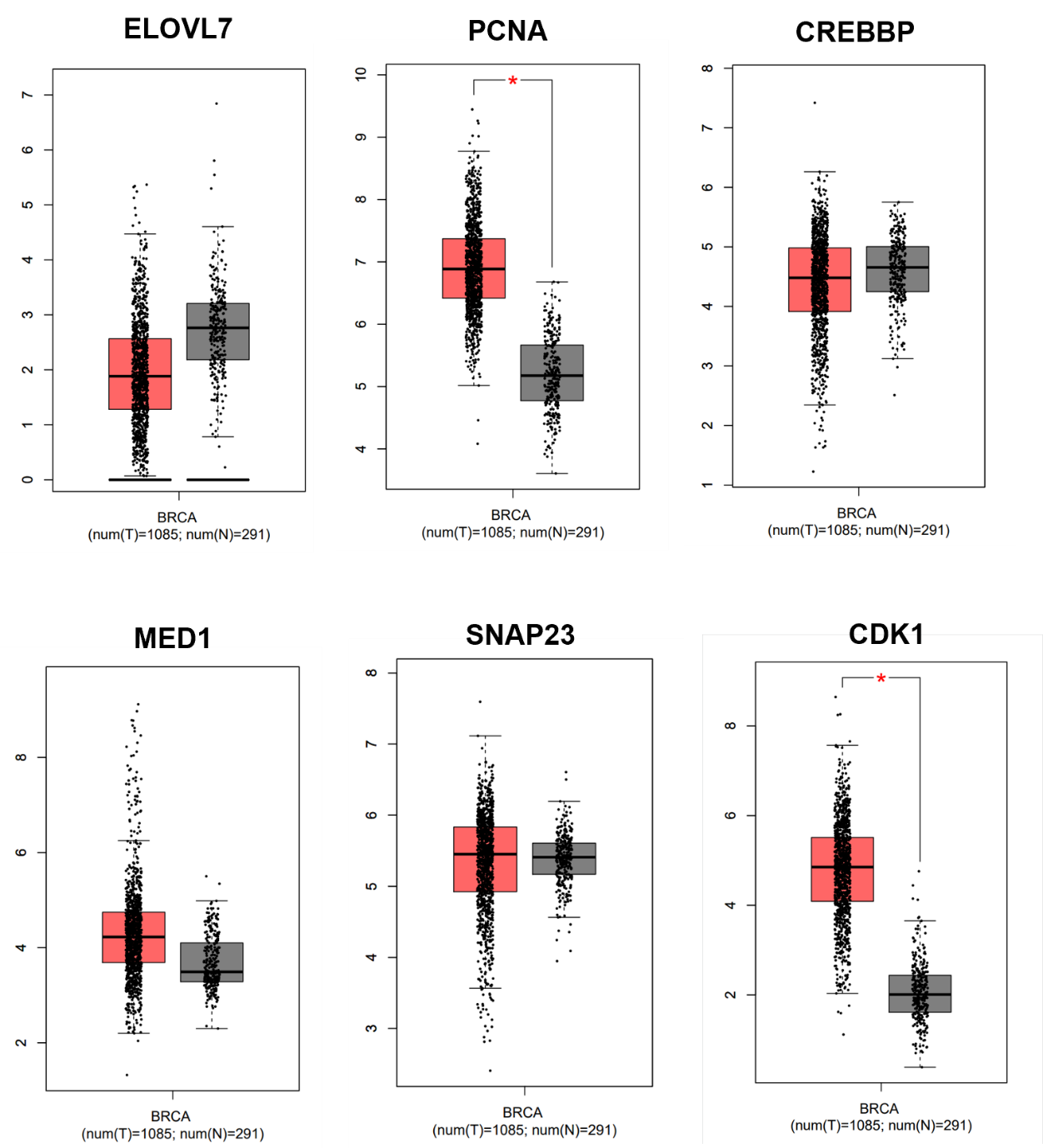


**Supplementary Figure 1.** The top six genes with betweenness centrality were expressed in normal people and Breast invasive carcinoma patients.
